# Challenges for Quality Assurance of Target Volume Delineation in Clinical Trials

**DOI:** 10.3389/fonc.2017.00221

**Published:** 2017-09-25

**Authors:** Amy Tien Yee Chang, Li Tee Tan, Simon Duke, Wai-Tong Ng

**Affiliations:** ^1^Department of Clinical Oncology, Pamela Youde Nethersole Eastern Hospital, Hong Kong, Hong Kong; ^2^Department of Clinical Oncology, University of Hong Kong, Hong Kong; ^3^Department of Oncology, Cambridge University Hospitals NHS Trust, Cambridge, United Kingdom

**Keywords:** target volume delineation variability, contouring guidelines, peer review, education program, clinical trial

## Abstract

In recent years, new radiotherapy techniques have emerged that aim to improve treatment outcome and reduce toxicity. The standard method of evaluating such techniques is to conduct large scale multicenter clinical trials, often across continents. A major challenge for such trials is quality assurance to ensure consistency of treatment across all participating centers. Analyses from previous studies have shown that poor compliance and protocol violation have a significant adverse effect on treatment outcomes. The results of the clinical trials may, therefore, be confounded by poor quality radiotherapy. Target volume delineation (TVD) is one of the most critical steps in the radiotherapy process. Many studies have shown large inter-observer variations in contouring, both within and outside of clinical trials. High precision techniques, such as intensity-modulated radiotherapy, image-guided brachytherapy, and stereotactic radiotherapy have steep dose gradients, and errors in contouring may lead to inadequate dose to the tumor and consequently, reduce the chance of cure. Similarly, variation in organ at risk delineation will make it difficult to evaluate dose response for toxicity. This article reviews the literature on TVD variability and its impact on dosimetry and clinical outcomes. The implications for quality assurance in clinical trials are discussed.

## Introduction

The last 20 years has seen the emergence of novel anticancer treatments which have the potential to improve clinical outcomes for patients. The standard method of evaluating such treatments is to conduct large scale multicenter clinical trials, often across continents. Radiotherapy is indicated for more than 50% of all cancer patients (1). Many oncology clinical trials, therefore, include radiotherapy within their treatment protocol even if the radiotherapy technique itself is not the subject of evaluation. Poor radiotherapy technique has been shown to be associated with inferior overall survival in many clinical trials; the benefit of any intervention in a clinical trial may, therefore, be compromised by suboptimal radiotherapy.

The radiotherapy quality assurance (RTQA) program was introduced to standardize radiotherapy across participating centers within a clinical trial. The RTQA program covers all aspects of the radiotherapy process including volume delineation, planning and delivery as well as infrastructure, equipment, personnel, and procedures. Several trial groups have reported that the implementation of RTQA procedures has enhanced protocol compliance and improved clinical trial outcome (2). However, the RTQA procedures in different clinical trials vary considerably making analysis and inter-trial comparisons to identify the most effective procedures difficult. Moreover, the cost of running a trial RTQA program is substantial, even more so with the introduction of advanced radiotherapy techniques.

Advanced radiotherapy techniques improve local tumor control and reduce treatment toxicity by delivering higher radiation doses to tumors while sparing adjacent normal tissue. Examples include intensity-modulated radiotherapy (IMRT), which allows the radiotherapy dose to be conformed to the target volume while sparing nearby organs at risk (OAR), and image-guided radiotherapy, which improves the precision of treatment delivery and allows smaller margins to be added to the target volume for delivery uncertainty (3). The benefit of these and other high precision techniques is critically dependent on optimal target volume delineation (TVD) by radiation oncologists as the steep dose gradients and reduced margins leave little room for error. There are numerous reports in the literature of suboptimal TVD, which can lead to fatal marginal recurrences due to geographical miss (4–8).

This article reviews the literature on TVD variability and its impact on dosimetry and clinical outcomes. The current methods for reducing TVD variability within and outside clinical trials and their limitations are discussed.

## Magnitude of TVD Variability

The delivery of radiotherapy treatment has long been subject to careful measurement and evaluation of the causes and magnitude of systematic and random errors. As a result, evidence-based strategies have been developed and universally adopted which have enabled radiotherapy delivery to approach millimeter precision.

In contrast, variability in TVD has not been evaluated with the same rigor. In 2016, Vinod et al. (9) published a systematic review of publications on uncertainties in TVD in radiation oncology. They identified 119 papers on TVD variability published between 2000 and 2014 covering the following clinical topics—breast, bladder, prostate, lung, esophagus, stomach, pancreas, liver, rectum, head and neck, brain, cervix, uterus, lymphoma, sarcoma, palliative radiotherapy, and OAR contouring. A number of studies focused on specific advanced radiotherapy techniques including image-guided brachytherapy (IGBT) for cervical cancer, stereotactic ablative body radiotherapy for lung cancer, and stereotactic radiosurgery for brain metastases.

All the studies showed considerable TVD variability between observers, often measured in centimeters. TVD variability was evident in all the volumes pertinent to radiotherapy planning as specified in ICRU Report 50 (10) published in 1978, i.e., the gross tumor volume (GTV), clinical target volume (CTV), and planning target volume (PTV).

Target volume delineation variability was seen among experienced radiation oncologists as well as trainees. There were also differences between different specialists [diagnostic radiologists, positron emission tomography (PET) physicians, neurosurgeons, orthopedic surgeons, gynecology oncologists, medical oncologists, hematologists, respiratory physicians] and disciplines (medical physicists and radiation therapists/radiographers). In one highly cited French study of GTV delineation in lung cancer (11), nine radiologists and eight radiation oncologists working in five different centers, classified as either “junior” or “senior” according to their professional experience, were asked to delineate the primary tumor and involved lymph nodes on the computed tomography (CT) images of 10 patients. The study showed that compared to radiation oncologists, radiologists tended to delineate smaller volumes and encountered fewer difficulties to delineate “difficult” cases. Junior doctors also tended to delineate smaller and more homogeneous volumes than their senior colleagues, regardless of their specialty, especially for “difficult” cases.

## Causes of TVD Variability

Despite the numerous papers on TVD variability within and outside clinical trials, very few have attempted to evaluate the causes of TVD variability in a systematic fashion.

Several studies have reported the impact of imaging modality on TVD variability. For example, a number of studies (12–14) showed that more consistent definition of the GTV in lung cancer can be obtained if the CT images were co-registered with 2-[18F]-fluoro-2-deoxy-d-glucose PET images. Similarly, there are studies showing more consistent definition of GTV and CTV of brain tumors on CT images co-registered with magnetic resonance images (MRI) (15). Image co-registration is now standard practice for both these tumor sites.

It is important to appreciate that reduced TVD variability seen on one imaging modality does not necessarily equate to this being a superior imaging modality. In a study on IGBT for cervical cancer (16), 23 gynecologic radiation oncology experts were asked to delineate the CTV on CT and MRI. There was a higher level of agreement of contours on CT despite MRI being universally recognized as the superior imaging modality. This probably reflects clinician unfamiliarity of MRI image interpretation for IGBT cervix planning where post-radiation changes can be a confounding factor.

It is commonly assumed that the major cause of intra-observer TVD variability is suboptimal image interpretation (17). However, other factors such as conceptual understanding of patterns of tumor spread and organ motion are equally important. In a study on definitive radiotherapy for cervical carcinoma (18), five radiation oncologists and two gynecologists independently contoured the CTVs for three patients. The study showed good consistency in outlined anatomical structures suggesting that image interpretation was not an issue. However, there was large inter-observer variability in CTV delineation with the ratio between largest and smallest volumes ranging between 3.6 and 4.9 for all observers. The ratio of common volumes to encompassing volumes ranged between 0.11 and 0.13 for the radiation oncologists, and between 0.30 and 0.57 for the gynecologists.

The TVD variability between gynecologists and radiation oncologists probably reflects different conceptual understanding of areas at risk of microscopic disease between the two specialties. The core skill for gynecologists is to remove the tumor with a small margin (usually 5 mm) with minimal disruption of surrounding tissue. In contrast, radiation oncologists irradiate large volumes of tissue to a relatively homogenous dose to minimize the risk of in-field and edge recurrences. The concepts of microscopic disease for these two specialties are, therefore, likely to be very different. This explanation could also account for the TVD variability between radiologists and radiation oncologists in the lung cancer study. Cancer radiologists are required to accurately define the tumor (avoiding both under and over estimation) to predict surgical resectability whereas the prime concern of radiation oncologists is to avoid missing the tumor. It is, therefore, easy to see why in difficult cases, some radiation oncologists would err on the side of caution and include areas of uncertainty in the GTV. Similarly, it is well recognized that junior doctors are less able to appreciate uncertainties than their senior colleagues, a phenomenon known as the Dunning Kruger effect based on Charles Darwin’s quote that “Ignorance more frequently begets confidence than does knowledge.”

Consistency and clarity of conceptual understanding is particularly important when new concepts are introduced. An example is the internal target volume (ITV), a concept first introduced in ICRU Report 62 published in 1999 (19). The ITV is defined as the CTV plus a margin taking into account uncertainties in size, shape, and position of the CTV within the patient. The margin for the ITV (called the internal margin) is distinct from the setup margin used for the PTV. However, in a survey of 50 radiation oncologists at a pelvic IMRT workshop (unpublished), 38% did not use the concept of the ITV in their daily practice, 30% incorporated the internal margin into the CTV, 26% incorporated the internal margin into the PTV, and only 8% contoured the ITV as a separate structure.

## Assessment of TVD Variability

The Vinod et al. review (9) reported that the number of imaging datasets in the studies on TVD variability varied from 1 to 132 with a median of 9, while the number of participants contouring ranged from 3 to 50 with a median of 7. There are no studies which have systematically analyzed the impact of number of imaging datasets or number of participants on TVD variability unlike the literature on setup accuracy. In those studies, where more than one case was used, the magnitude and direction of TVD variability varied considerably between cases reflecting the variation in patient anatomy and tumor topography.

There was also a wide range of methods used to assess TVD variability. A volume metric (volume measurements, volume ratios) was most consistently reported across most studies. Measures of overlap (concordance index, discordance index, dice similarity coefficient) were also frequently reported. Comparisons were usually measured against a reference contour. The definition of a reference contour varied from the contour of a recognized expert to a consensus contour with multiple observers or a Simultaneous Truth and Performance Level Estimation (STAPLE) contour (20) (STAPLE is the probabilistic estimate of the “true” volume generated from all observers). All these methods have an inherent deficiency in that they do not provide any information on the location of any discrepancies or their clinical significance.

## Dosimetric Impact of TVD Variability

Vinod identified only 25 (21%) studies which evaluated the impact of variability in target and OAR contouring on dosimetry (9). Thirteen studies evaluated the dosimetric impact of target volume variability; it was interesting that three of these studies found no significant impact on PTV dose coverage. Ten studies also evaluated the impact of target volume variability on OAR doses; of these, eight studies found a significant impact on OAR dose–volume histograms (DVH). Twelve studies examined the impact of variability in OAR volume delineation; eight of these studies found statistically significant differences in OAR doses.

Vinod classified the analysis of the dosimetric impact of TVD variability into three broad methods. The first method involved a reference plan (usually the treatment plan or a plan optimized to a reference or expert contour) being applied to the volumes of many observers. This technique was used by Hellebust et al. (4) to study the dosimetric impact of contouring variations on a group of patients treated with IGBT for cervix cancer. They found that that the dose to the GTV and high-risk CTV (HR-CTV) had the smallest variation compared to the dose to the intermediate risk CTV (IR-CTV). This is perhaps not surprising as the IR-CTV is a new and complex concept, first introduced in 2005, which requires the clinician to integrate the CTV at the time of brachytherapy (BT) with the GTV at diagnosis. For OAR, the dose effect was largest for the sigmoid colon which again illustrates the greater uncertainty in defining this organ compared to the rectum and bladder. Overall, TVD variability resulted in a deviation of up to 5 Gy to the HR-CTV and up to 3 Gy for OAR.

The same method was used by Loo et al. (5) to investigate the dosimetric impact of variability in OAR contouring for head and neck IMRT. Four radiation oncologists and three radiologists delineated the parotid gland on the CT datasets of 10 patients with oropharyngeal carcinoma treated with parotid-sparing IMRT. The DVH for each study contour was calculated using the IMRT plan actually delivered for that patient and was compared with the original DVH obtained when the plan was used clinically. The mean parotid dose achieved during actual treatment was within 10% of 24 Gy for all patients. However, using the study contours, the mean parotid dose was within 10% of 24 Gy for only 53% of volumes by radiation oncologists and 55% of volumes by radiologists. The parotid DVH of 46% of the study contours were sufficiently different from the clinical DVH, such that a different IMRT plan would have been produced.

The second method as identified by Vinod is the converse of method one. In this method, the plans generated from many observer volumes are assessed for resultant dosimetry on a reference volume. This method was used in the INTERLACE study on IMRT for cervix cancer (6). No plan generated from the observer volumes was found to achieve the optimal gold standard PTV (GS-PTV) coverage; on average, the resultant dose (V95%, D95%) was 10–20% lower. The GS-PTV volume outside the 95% isodose ranged from 83 to 458 cc. A qualitative assessment showed the most common anatomical areas not covered by the 95% isodose were vagina, obturator, and nodal regions such as external iliac nodes.

In the first two methods, there is an assumption that the reference plan is “correct” and based on a “gold standard” volume which is again correct. If the reference plan is based on a volume that is an outlier compared to the contours being analyzed, the systematic differences measured may be amplified. In contrast, the third method involves a comparison of all plans applied to all contours without a reference. A plan is optimized to a particular delineated volume and then applied to all other volumes to assess dosimetry. This is then repeated for each observer’s volume. This allows for the most in-depth comparison of dosimetry relating to TVD variability but is also the most resource-intensive.

The third method was used in a lung cancer study by Van de Steene et al. (21) in which five clinicians were asked to define the GTV (tumor and lymph node) on the planning CT scans of eight patients. For each volume, a standard conformal treatment plan comprising two pairs of opposed antero-posterior and lateral beams were created. The study reported inter-observer variation in the dimensions of the primary tumor of up to 4.2 (transverse), 7.9 (cranio-caudal), and 5.4 cm (antero-posterior). The variation in the extreme extensions of the GTV (tumor and lymph nodes) ranged from 2.8 to 7.3 cm. After common review, only 63% of involved lymph node regions were delineated by the clinicians (i.e., 37% were false negative). The probability (in the population of all conformal plans) of irradiating at least 95% of the GTV with at least 95% of the nominal treatment dose decreased from 96% for a matched plan (i.e., a plan created for that GTV volume) to 88% for an unmatched plan.

The authors suggested four possible causes for the large inter-observer variation—problems with methodology including definitions and concepts (e.g., definition of GTV to exclude atelectasis, definition of involved lymph nodes based on size, contouring of individual lymph nodes, or lymph node regions), difficulty differentiating between tumor and benign pathology (e.g., atelectasis), difficulty differentiating between tumor and normal structures, and lack of knowledge of anatomy. Interestingly, they also concluded that only the minority of the issues could be resolved objectively.

## Clinical Impact of TVD Variability

There are no studies which have assessed the direct impact of TVD variability on clinical outcome.

Peters et al. (8) retrospectively analyzed 780 patients in the Trans-Tasman Radiation Oncology Group 02.02 (TROG 02.02) HeadSTART trial in head and neck cancer and found that patients whose radiotherapy plans failed trial quality assurance (12% overall) had poorer survival and loco-regional control compared to the those with protocol-compliant plans [2-year overall survival (OS) 50 vs. 70%, *p* < 0.001, 2-year loco-regional control 54 vs. 78%, *p* < 0.001]. However, incorrect volume delineation was a feature in only 25% (24/97) non-compliant plans.

A number of studies have modeled the potential impact of TVD variability. Van de Steene et al (11) estimated the impact of GTV delineation variability on tumor cure probability (TCP). Across all plans, the mean TCP decreased from 51% for a matched plan (i.e., a plan created for that GTV volume) to 42% for an unmatched plan (i.e., a plan created for another GTV), a difference of 9%. The mean range in TCP across the eight patients was 2% (maximum range 5%) for matched plans compared to 14% (maximum 31%) for unmatched plans. They also estimated the normal tissue complication probabilities for different OAR but this analysis was of limited value as the plans used were 4-field boxes which would not have been used clinically.

Jameson et al. (7) also modeled the impact of GTV delineation variability on TCP and equivalent uniform dose (EUD) in lung cancer. Three radiation oncologists contoured the GTV on the planning CT, the diagnostic PET–CT and the radiotherapy planning PET–CT for seven patients. An optimized plan with 3–5 conformal beams was created for each volume. The SD of the volumes across all seven patients ranged from 39 to 419 cc. However, the SD of the EUD was ≤1 Gy in four of the seven patients (range 0.09–21.2 Gy). Similarly, the SD of the TCP was negligible (0–1%) in four of the seven patients (range 0–22%). Contouring variations in the lateral dimensions had the greatest impact on EUD and TCP.

## Minimizing TVD Variability in Routine Practice

Several interventions have been developed to reduce inter-observer TVD variability. These have been reviewed in another publication by Vinod et al. (21).

### Contouring Guidelines and Atlases

The most common method for reducing TVD variability within and outside clinical trials is probably the use of consensus contouring guidelines and/or atlases (22, 23). Lobefalo et al. (24) evaluated the benefit of a contouring guideline on consistency of TVD in a study of rectal cancer. Four radiation oncologists contoured the CTV on 10 patients before and after the introduction of a shared guidelines. The Agreement Index improved from 0.57 (pre-guideline) to 0.69 (post-guideline). The unmatched PTV coverage improved from 93.7 ± 9.2 to 96.6 ± 4.9% for 3D conformal radiotherapy and 86.5 ± 13.8 to 94.5 ± 7.5% for a volumetric modulated arc radiotherapy (VMAT) technique. This suggests that the dosimetric impact of inter-observer variation is more pronounced for advanced radiotherapy techniques.

Eminowicz et al. (22) from the INTERLACE trial reported the reduction of inter-observer contouring variation and increased protocol adherence after introduction of an atlas. They analyzed seven key guidelines for target volume contouring in cervical cancer and identified 11 common areas of variation. A pictorial atlas was then derived to illustrate a consistent delineation method for these areas. The average proportion of outlines (of 4; primary CTV, nodal CTV, bladder, rectum) complying to the protocol improved from 1.8/4 to 2.7/4 with atlas use.

While contouring guidelines are undoubtedly invaluable in making TVD more consistent, they can also be a source of variability if different groups produce conflicting guidelines for the same tumor site or anatomical region. For example, the GYN consortium consensus guidelines for CTV delineation for IMRT for cervix cancer defines the lateral border of the parametrium as the medial edge of internal obturator muscle/ischial ramus (i.e., lateral to the pelvic vessels) whereas the EMBRACE-II guidelines define this border as the medial edge of internal iliac and obturator vessels. Similarly, the inferior border of the pre-sacral nodes has been defined as S2 in gynecological guidelines (23, 25), S3 in prostate guidelines (26, 27) and bottom of the coccyx in anal guidelines (28, 29). It is easy to see how a clinician used to contouring in a particular way will continue to do so in a clinical trial regardless of the protocol specification.

### Multi-Modality Imaging

Improved imaging, e.g., use of intravenous contrast, optimal window settings, and multi-modality imaging, is an intuitive way to improve TVD consistency. In the Vinod et al. review (9), there were more published studies using this method than all other methods combined. However, results have been mixed and 9 of the 31 studies reviewed did not demonstrate a statistically significant reduction in TVD variability. It appears that interpretation of the additional imaging modality and image co-registration are sources of error in themselves.

### Auto-Contour Provision

A few studies have reported improved TVD consistency from clinicians editing an auto-contour compared to manual delineation (21). However, if the auto-contour contains an error, then this is more likely to be transmitted through the manual editing process as a systematic error. The majority of auto-contouring software in clinical use utilize atlas-based segmentation which always requires manual review and adjustment due to the wide variation in normal and post-treatment anatomy. Machine learning techniques hold promise for increasing accuracy and reducing the burden of user editing as discussed in a review by Sharp et al. (30).

### Contouring Workshops and Educational Programs

Several publications have reported the benefit of contouring workshops on reducing TVD variability. An example is an International Atomic Energy Agency study over a 1-year period involving 11 pairs of clinicians comprising a radiation oncologist and a nuclear medicine physician (31). Training consisted of lectures, contouring practice, and group and individualized feedback. Following the first training, overall concordance indices for three repeated cases increased from 0.57 ± 0.07 to 0.66 ± 0.07. After further training, overall concordance indices for another three repeated cases further increased from 0.64 ± 0.06 to 0.80 ± 0.05 (*p* = 0.01).

Contouring workshops are a popular method for teaching TVD but they have several limitations. In most cases, improvement is measured by re-contouring on the same cases and it is difficult to ascertain whether learning is transferred to different cases with different patient anatomy and tumor topography. The number of participants is limited by logistics and cost.

Recent advances in technology such as web-enabled video conferencing and interactive software have enabled both live and offline educational interventions to reach across geographical boundaries. An example is the FALCON program (Fellowship in Anatomic delineation and Contouring), offered by the European Society for Radiotherapy & Oncology (32). However, online workshops will face the same pedagogical issues as live ones.

A few contouring tools have been developed to support self-learning TVD programs. These tools offer delineation practice often with provision of a reference volume and/or automated feedback. These programs are in their infancy and their utility remains to be established. Issues include difficulty in defining a reference volume given the extent of disagreement in TVD among experts, challenges for user engagement and outdated internet access particularly in hospitals.

### Peer Review

Peer review involves the review of aspects of radiotherapy treatment by two or more radiation oncologists, or another specialist such as a radiologist. It may cover indications for treatment, treatment approach, volume delineation, planning directives, evaluation of plan quality and/or treatment verification. The American Society for Radiation Oncology has identified TVD as the first priority for peer review due to the heterogeneity in contouring and its impact on the rest of the radiotherapy process (33).

Multiple audits of peer review have identified that a proportion of radiotherapy treatments require significant alteration. In an early study (34), 3,052 cases were reviewed over 8 years of which 4.1% were “not approved.” More recently, Mackenzie et al. (35) presented a prospective audit of peer review meetings in breast, head and neck, and lung cancer. Overall 9% of treatments required alteration before the first or next fraction of radiotherapy, although this varied significantly across the tumor sites (1–16%). A study by Dimigen et al. (36) reported that involving a radiologist in weekly QA meetings resulted in a significant change in management in 6% of cases.

Multiple professional organizations now advocate peer review as an important component of safe and effective radiotherapy. However, there are significant barriers to its implementation including a lack of personnel, dedicated time and facilities, and a reluctance of clinicians to invite scrutiny, especially across institutions. Given its cost and resource implications, rigorous research to evaluate its benefit is urgently needed. Technologies which allow large scale remote assessment of contours would be hugely advantageous.

## Minimizing TVD Variability in Clinical Trials

The process for RTQA of TVD in clinical trials may involve one or more of the following (37):

- A benchmark case—the participating institution is asked to delineate radiotherapy volumes on one or more standardized cases according to the protocol.
- A dummy run—the institution uploads the datasets of one or more of their patients treated locally for central review.
- Individual case review—during the course of the trial, some or all of the patients’ radiotherapy datasets will be requested for prospective or retrospective central review.

Most of the reports on RTQA for TVD have used benchmark cases. An example is the INTERLACE study on IMRT for cervix cancer. The principal investigators (PIs) of participating centers were asked to contour the CTV on two cases with different FIGO stages. 21 outlines were compared for case 1 and 22 for case 2. The delineated volumes ranged from 340 to 676 cc for case 1 and 458 to 806 cc for case 2. The direction of the maximum variation was different in the two cases.

The EMBRACE-I study on IGBT for cervix cancer is an example of RTQA based on a dummy run (38). Each center was asked to upload a “good response” case and a “poor response” case for central review. The review was qualitative with one physician reviewing all the external beam radiotherapy (EBRT) contours and three other physicians reviewing the BT contours. Out of 30 submitting centers, 13 had major inconsistencies in BT contouring while 11 had major inconsistencies in EBRT contouring. Centers with experience in IGBT (>30 cases) performed better than those with limited experience.

Retrospective individual case review was reported by the SCALOP trial in pancreatic cancer (39). The chief investigator and a radiologist contoured the GTV on the 60 of 74 patients who received radiotherapy in the study (12 patients had planning CTs which were deemed to be of insufficient quality for re-contouring) and compared their gold standard contours with the treating clinicians’ contours using the Jaccard conformity index and geographical miss index. The median geometric indices for GTV and PTV seen in on-trial patients were better than the pre-trial benchmark case, suggesting that overall, quality of tumor delineation was acceptable and that the pre-trial RTQA may have enhanced the quality of tumor delineation within the main trial. However, tumor was completely missed in one patient, and ≥50% of the tumor was missed in three cases. The authors reported that patients with Jaccard conformity index for GTV ≥ 0.7 had 7.12 (95% CIs: 1.83–27.67, *p* = 0.005) higher odds of progressing by 9 months in multivariate analysis, which is counter-intuitive.

## Discussion

Our review has found that although there are numerous publications reporting considerable TVD variability within and outside clinical trials, there are very few which have investigated the causes of the variability or its impact on actual clinical outcomes. The limited data on outcomes are conflicting with modeling papers suggesting different impact on TCP in different patterns which is perhaps not surprising. The one paper which correlated TVD variability with outcomes showed that higher concordance with the gold standard contours actually worsens outcome. All the data to date suggest that the relationship between TVD variability and outcome is not straightforward and further research is required. Similarly, several educational strategies have been put forward to minimize TVD variability but there is little systematic research into the effectiveness of the strategies and more importantly, whether learning is retained.

The problem is particularly acute for clinical trials due to the requirement to assess clinicians from many participating centers, in dispersed locations. The logistics are such that most clinical trials limit their RTQA process to the PIs who are probably the most likely to contour correctly. Similarly, most RTQA is based on 1 or 2 carefully chosen benchmark cases which does not take into account patient anatomy and difficult topography. The assessment process is usually subjective and there may be a conflict of interest for the central review team to “pass” centers in order to increase trial recruitment.

In 2010, the Global Clinical Trials RTQA Harmonization Group (GHG) (40) was established to

- collate, homogenize and distribute information regarding the RTQA standards of clinical trial groups,
- provide a platform for prospective discussions on new RTQA procedures, software tools, guidelines and policies of trial groups,
- provide a framework to endorse existing and future RTQA procedures and guidelines across various trial groups.

The aim is to increase cooperation between trial groups internationally and facilitate the exchange and interpretation of RTQA data.

Perhaps a neglected opportunity in clinical trials is the potential to use RTQA content for systematic education. This strategy has been adopted in the EMBRACE-II study of IMRT and IGBT in cervix cancer (www.embracestudy.dk). In addition to workshops and annual update meetings, the study has set up an online continuous education program for all study participants. The program includes a number of educational resources not commonly available in clinical trials such as training contouring cases and quizzes. The quizzes in particular have been popular with participants and have identified gaps in knowledge and participant comprehension of the protocol. This has enabled the trial management group to develop targeted learning resources which should hopefully improve protocol compliance. The aim is to eventually make these resources available to non-trial participants as well.

## Conclusion

Target volume delineation variability is a significant problem in radiotherapy both within and outside clinical trials. More research is required to evaluate the causes of variability and its impact on dosimetry and clinical outcome.

## Author Contributions

AC: draft outline and final manuscript. LT and SD: revision of some sections of the manuscript and organization of references. W-TN: revision and review of the manuscript.

## Conflict of Interest Statement

The authors declare that the research was conducted in the absence of any commercial or financial relationships that could be construed as a potential conflict of interest.
